# Validating a Stability Indicating HPLC Method for Kinetic Study of Cetirizine Degradation in Acidic and Oxidative Conditions

**Published:** 2013

**Authors:** Effat Souri, Ali Hatami, Nazanin Shabani Ravari, Farhad Alvandifar, Maliheh Barazandeh Tehrani

**Affiliations:** *Department of Medicinal Chemistry, Faculty of Pharmacy and Pharmaceutical Sciences Research Center, Tehran University of Medical Sciences, Tehran, Iran. *

**Keywords:** Cetirizine, High-Performance Liquid Chromatography (HPLC), Degradation, Kinetics, Stability

## Abstract

A stability indicating High-Performance Liquid Chromatography (HPLC) method was validated and used to study the degradation of cetirizine dihydrochloride in acidic and oxidative conditions. The separation was carried out on a Symmetry C_18_ column and a mixture of 50 mM KH_2_PO_4 _and acetonitrile (60:40 v/v, pH = 3.5) was used as the mobile phase. The method was linear over the range of 1-20 μg/mL of cetirizine dihydrochloride (r^2^ > 0.999) and the within-day and between-day precision values were less than 1.5%. The results showed that cetirizine dihydrochloride was unstable in 2 M HCl and 0.5% H_2_O_2_. The kinetics of the acidic degradation showed a pseudo-first-order reaction in the temperature range of 70-90°C. In addition, the kinetics of hydrogen peroxide mediated degradation was pseudo-first-order in the temperature range of 50-80°C.

## Introduction

**Figure 1:**
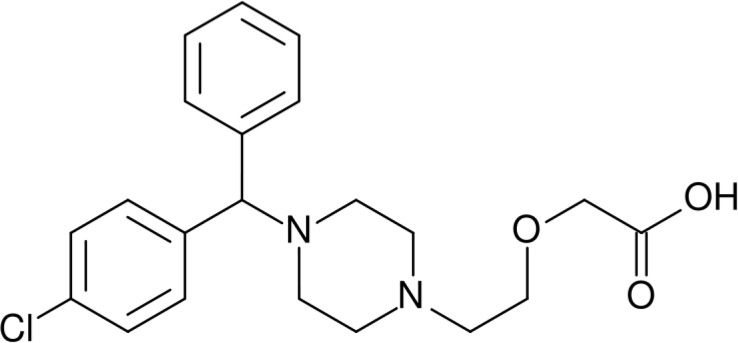
Chemical structure of cetirizine

Cetirizine dihydrochloride, chemically known as (RS)-2-[2-[4-(4-chlorophenyl) phenylmethyl] piperazin-1-yl] ethoxy] acetic acid dihydrochloride (Figure 1), is a non-selective antagonist of H_1_-receptor. Cetirizine dihydrochloride is a potent and non-sedating antihistamine belongs to the piperazine class of second generation of antihistamines. Cetirizine dihydrochloride is used for symptomatic treatment of allergic conditions including seasonal rhinitis and chronic urticaria (1). 

Literature survey showed several reported High-Performance Liquid Chromatography (HPLC) (2-9), capillary zone electrophoresis (10) or spectrophotometric (2, 11) methods for determination of cetirizine dihydrochloride in pharmaceutical dosage forms either alone or in combination with other drugs. In addition, a potentiometric method is reported in BP for determination of cetirizine dihydrochloride in bulk powder (12). 

According to the ICH guidelines, the stability of drug substances should be studied in different conditions. There are some reports in the literature about the stability of cetirizine dihydrochloride in different conditions. According to Makhija *et al. *(13), no degradation was observed for cetirizine in combination with pseudoephedrine under acidic (1 M HCl) or basic (1 M NaOH) conditions at 70°C after 2 h. On the other hand cetirizine was unstable under oxidative conditions. By using 0.1 M HCl, 0.1 M NaOH or 1% H_2_O_2_ at 80°C for 10 h, it has been concluded that cetirizine dihydrochloride was stable in basic condition but unstable in acidic or oxidative conditions (4). There is another report regarding the stability of cetirizine dihydrochloride under 0.5 M HCl or 0.5 M HCl at 80°C after 4 h (7). Using 1 M HCl or 30% H_2_O_2_ decomposition of cetirizine dihydrochloride was observed after 12 h at 80°C (8). To the best of our knowledge, these reports are at a descriptive level and there is no research published in the literature in regard to the kinetics of degradation of cetirizine dihydrochloride under acidic, basic or oxidative conditions.

The aim of this study was to validate an HPLC method according to the ICH guidelines to be performed for kinetic study of the degradation of cetirizine dihydrochloride under acidic, basic and oxidative degradation processes.

## Experimental

*Materials*

Cetirizine dihydrochloride was from Auctus Pharma Limited (Unit-II), India (Batch No, CZH-10 01 001). All the HPLC grade solvents and analytical grade chemicals were purchased from Merck (Darmstadt, Germany). HPLC grade water was obtained by a Milli-Q purification system (Millipore, Milford, MA, USA).

*Instrumentation*

A Waters HPLC system consisted of an isocratic pump (Model 515), an autosampler (Model 710 plus) and a variable UV-vis detector (Model 480) was employed. The HPLC data were processed by using a multi-channel Chrom and Spec software for chromatography, version 1.5 x.

*Chromatographic conditions*

Chromatographic Conditions was partly based on the method reported by Zaater *et al. *(14). A Symmetry^®^ C_18_ 5 μm column (4.6 mm × 150 mm, Waters) and a mixture of KH_2_PO_4_ 50 mM and acetonitrile (60:40 v/v, pH = 3.5) at a flow rate of 1 mL/min were used. The mobile phase was prepared daily and degassed by filtration through a 0.45 μm Teflon membrane filter (Millipore, Milford, MA, USA) and sonication for 10 min. The wavelength for UV-detection was 230 nm and all determinations were performed at ambient temperature.

*Standard solution*

Stock standard solution of cetirizine dihydrochloride was prepared by dissolving an accurately weighed portion of drug in water to reach a concentration of 5000 μg**/**mL. The stock standard solution was kept at 4°C protected from light. Standard solutions for calibration curve (1, 2, 5, 10, 15 and 20 μg/mL) were prepared daily by appropriate dilution of stock standard solution with mobile phase to reach the desired concentration.

*System suitability*

System suitability parameters of the HPLC method were assessed by six replicate injections of a solution of cetirizine dihydrochloride (10 μg/mL in mobile phase) to the HPLC system. The coefficient of variations for the peak areas and retention times were calculated.

*Linearity*

Six series of cetirizine dihydrochloride solutions in mobile phase at the concentrations of 1, 2, 5, 10, 15 and 20 μg/mL were prepared and injected to the HPLC system. The calibration curves were constructed by plotting the measured peak areas of each concentration against the corresponding concentration and the statistical analysis was performed.

*Precision and accuracy*

Cetirizine dihydrochloride solutions at three different concentration levels within the calibration range (1, 5 and 20 μg/mL of mobile phase) was injected to the HPLC system in triplicate. The concentration of each solution was measured using a calibration curve in the range of 1-20 μg/mL. The within-day precision and accuracy was calculated. The same method was performed during three separate days to find out the between-day precision and accuracy.

*Stability*

The stability of cetirizine dihydrochloride stock solution was checked after storing at 4°C for 1 week.

*Application of the method*

Twenty cetirizine dihydrochloride tablets were weighed and ground into fine powder using a glass mortar and pestle. An accurately weighed portion of powder equivalent to 10 mg cetirizine was transferred to a 100 mL volumetric flask and 50 mL of mobile phase was added. After 15 min sonication the volumetric flask was adjusted to the volume with the same solvent. The solution was injected to the HPLC system after filtration through a 0.45 μm polypropylene syringe filter (Teknokroma, Spain) and ten times dilution. The drug concentration was determined in comparison with the same concentration of a standard solution of cetirizine dihydrochloride.

*Recovery*

To find out the relative recovery of cetirizine from dosage forms, an accurately weighed amount of tablet powder equal to 50% of one tablet was spiked with cetirizine dihydrochloride standard solution in a 100 mL volumetric flask. The same procedure for determination of drug dosage form was performed. The peak area of the triplicate injections was compared by a standard solution of cetirizine dihydrochloride with the same concentration level and the relative recovery was calculated.

*Kinetic investigation of cetirizine degradation*

For acidic degradation, 1 mL of stock standard solution of cetirizine dihydrochloride (5000 μg/mL) was transferred into a 10 mL volumetric flask and 2 M HCl was added to volume. The flask was placed in a dry air oven (Melag, Germany) at four different temperatures (70, 80, 85 and 90°C). At specified time intervals, 500 μL of the solution was transferred to a 10 mL volumetric flask and after neutralization with sodium hydroxide, adjusted to the volume by mobile phase. The resulting solution was injected to the HPLC system and the peak area of cetirizine was compared with a freshly prepared standard solution. The percentage of remained cetirizine was calculated and plotted against time. Each experiment was repeated three times at each temperature.

For basic degradation, the same experiment was performed using 5 M NaOH and for neutralization, a solution of hydrochloric acid was used.

Oxidative degradation was also performed using the same procedure by using 0.5% hydrogen peroxide at four different temperatures (50, 60, 70 and 80°C).

## Results and Discussion

*Chromatographic conditions*

**Figure 2:**
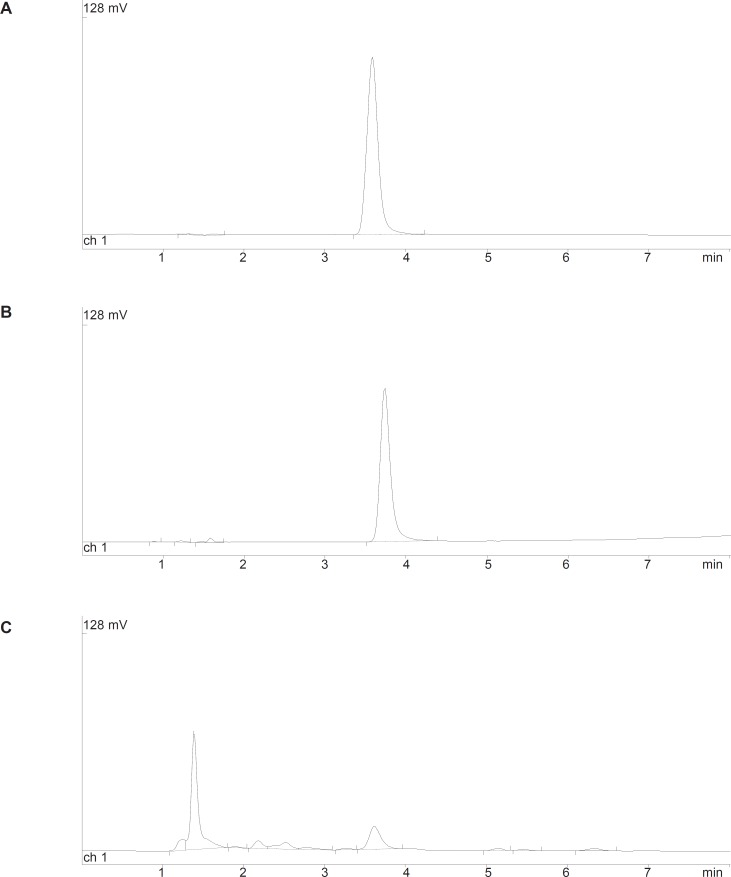
Typical chromatograms obtained from stability studies of cetirizine DIhydrochloride. A: cetirizine DIhydrochloride standard solution (25 μg/mL); B: cetirizine dihydrochloride solution in 2 M HCl at 70ºC after 72 h; C: cetirizine dihydrochloride solution in 0.5% H_2_O_2_ at 70ºC after 3 h.

As mentioned in the experimental section, the chromatographic conditions were partly based on a previously published method (14). Using a Symmetry C_18_ column and a mixture of KH_2_PO_4_ 50 mM and acetonitrile (60:40 v/v, pH = 3.5), good peak shape without tailing and interferences from degradation products in acidic or oxidative conditions, was observed (Figure 2a). The system suitability parameters showed in Table 1 were within the acceptable criteria.

**Table 1:** System suitability parameters for chromatographic conditions

**Parameters**	**Found**	**Acceptable limits**
**USP theoretical plates (n = 6)**	3500	N > 1500
**USP tailing factor (n = 6)**	1.08	T < 1.5
**Repeatability (t**_R_ **) (n = 6)**	0.23	RSD < 1%
**Repeatability (peak area)(n = 6)**	0.50	RSD < 1%

t_R_: Retention time (min); N: Theoretical plate; T: Tailing factor; RSD: Relative Standard Deviation

*Linearity*

The HPLC method was linear over the concentration range of 1-20 μg/mL of cetirizine dihydrochloride. According to the data shown in Table 2, acceptable linearity with r^2^ > 0.999 was observed. The limit of quantification (LOQ) with CV < 1.5% was found to be 1 μg/mL. The limit of detection (LOD) based on S/N ratio of 3 was found to be 0.2 μg/mL.

**Table 2:** Statistical data of calibration curves of cetirizine dihydrocholride (n = 6)

**Parameters**	**Results**
Linearity range	1-20 µg/mL
Regression equation	y = 39.49x - 8.39
Standard deviation of slope	0.15
Relative standard deviation of slope (%)	0.39
Standard deviation of intercept	0.42
Correlation coefficient (r^2^)	0.9994

*Precision and accuracy*

The results of the precision and accuracy of the method at three different concentration levels are demonstrated in Table 3. The intermediate precision was also studied by comparison of the assay results for cetirizine tablets by two analysts using two different HPLC systems. The CV values did not exceed 2%.

**Table 3:** Precision and accuracy of the method for determination of cetirizine dihydrochloride (Three sets for 3 days).

**Added (** µ**g/mL)**	**Recove** **r** **ed** **(mean ±** **SD) (**µ**g/mL)**	**CV(%)**	**Er** **r** **or(%)**
**W** **ithin-day (n =** **3)**			
1.00	1.01 ± 0.01	1.13	1.00
5.00	5.00 ± 0.03	0.62	0.00
20.00	19.98 ± 0.16	0.80	- 0.10
**Between-day (n =** **9)**			
1.00	1.01 ± 0.01	1.45	1.00
5.00	5.00 ± 0.04	0.88	0.00
20.00	19.90 ± 0.16	0.81	- 0.45

*Relative recovery*

The mean recovery of cetirizine dihydrochloride calculated by standard addition method to tablet powder was about 99% and no interfering peaks from excipients were observed.

*Solution stability*

The stock standard solution of cetirizine dihydrochloride in water was stable over a period of one weak at 4°C with a recovery of about 99.5%.

*Analysis of pharmaceutical product*

The cetirizine tablets were determined by using the proposed method. The results showed good agreement with the labeled amount (10.04 ± 0.06 mg per tablet).

*Degradation studies*

In a preliminary study the degradation of cetirizine dihydrochloride was studied in 0.1 M HCl, 0.1 M NaOH and 1% H_2_O_2_ at 80°C according to the reported method of Jaber *et al. *(4). The degradation in 1% H_2_O_2_ was too fast and no degradation was observed in 0.1 M NaOH. Subsequent studies were repeated by using higher strength of NaOH and HCl and lower strength of H_2_O_2_.

Cetirizine dihydrochloride was more stable in basic condition even under higher strength of NaOH (1 M, 2 M and 5 M). In fact, a very small loss of peak area was observed after long exposure to 5 M NaOH at 90°C up to 48 h.

*Kinetics of degradation in acidic condition*

**Figure 3:**
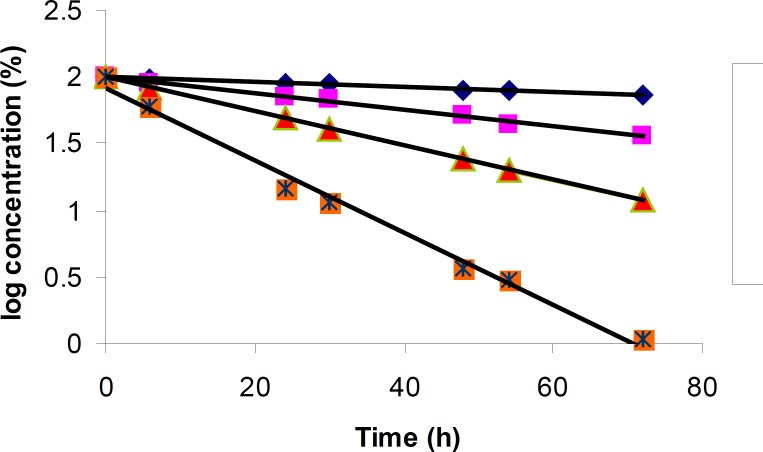
Pseudo-first-order plots for the degradation of cetirizine dihydrochloride in 2 M HCl at various temperatures using HPLC method. Key; Ct, percent remained cetirizine dihydrochloride at time t, and C_0_, percent cetirizine dihydrochloride at zero time

The degradation of cetirizine dihydrochloride was investigated in acidic condition using 2M hydrochloric acid. The drug was found to degrade in this condition at different temperatures with a same pattern (Figure 2b). A regular decrease in cetirizine dihydrochloride concentration was observed with increasing time. A linear relationship was observed by plotting the 1og of residual percent cetirizine dihydrochloride versus time (Figure 3) which indicates a pseudo-first-order kinetics according to the following equation: 

log C_t_ = log C_0 _- kt / 2.303

Here, Ct is the percent remained cetirizine peak area, C_0_ is the initial percent of cetirizine (100%), k is the apparent first order rate constant with a negative sign and t is the time. Good correlation coefficient for all plots in different temperatures was observed (CV > 0.99). The values for the apparent first order rate constants and the half-lives are presented in Table 4. The rate of degradation was decreased with temperature reduction.

**Table 4:** Degradation equation, apparent rate constant (k) and half-life (t_1/2_) for cetirizine dihydrochloride in 2 M HCl

**Temperature (**°**C)**	**Equation**	**r** ^2^ **-value**	**k (h** ^-1^ **)**	**t**_1/2 _ **(h)**
70	y = - 0.002x + 2.003	0.999	0.005	138.6
80	y = - 0.006x + 1.998	0.992	0.014	49.5
85	y = - 0.013x + 2.000	0.994	0.030	23.1
90	y = - 0.027x + 1.917	0.991	0.062	11.2

**Figure 4:**
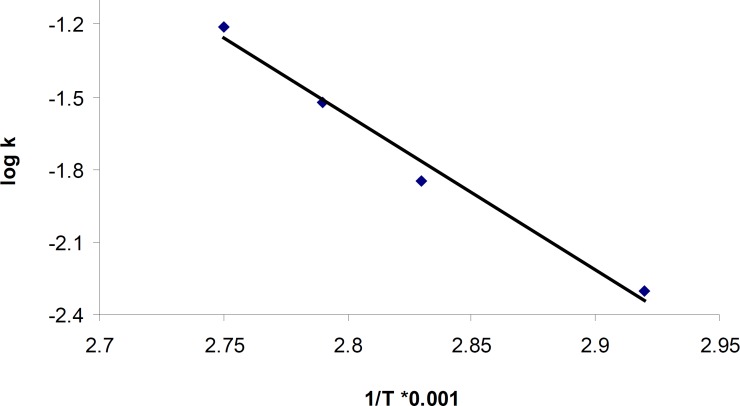
Arrhenius plot for degradation of cetirizine dihydrochloride in 2 M HCl

Based on the Arrhenius relationship in the temperature range of 70-90°C (Figure 4), the following equation was used to calculate the activation energy for the acid-mediated degradation process of cetirizine which was about 121.8 KJ/mole.

*K=Ae* ^_Eact/RT^

Here, k is the apparent first order rate constant. A is the pre-exponential factor, Eact is the activation energy and T is the temperature.

No significant degradation was observed at lower temperatures. So, the kinetics of degradation could not be monitored in these temperatures.

*Kinetics of degradation in oxidative condition*

The degradation of cetirizine dihydrochloride was too fast in 1% H_2_O_2_ medium. Using 0.5% H_2_O_2_ the degradation reaction could be followed at four different temperatures (50, 60, 70 and 80°C). Degradation pattern was similar in all conditions and a few small new peaks were detected in the chromatogram (Figure 2c).

**Figure 5:**
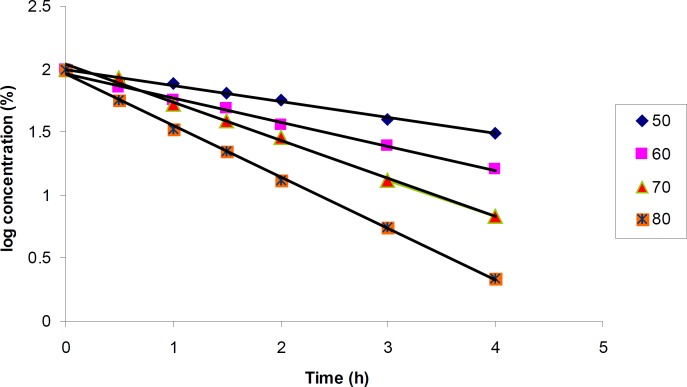
Pseudo-first-order plots for the degradation of cetirizine dihydrochloride in 0.5% H_2_O_2_ at various temperatures using HPLC method. Key; Ct, percent remained cetirizine dihydrochloride at time t, and C_0_, percent cetirizine dihydrochloride at zero time

**Figure 6:**
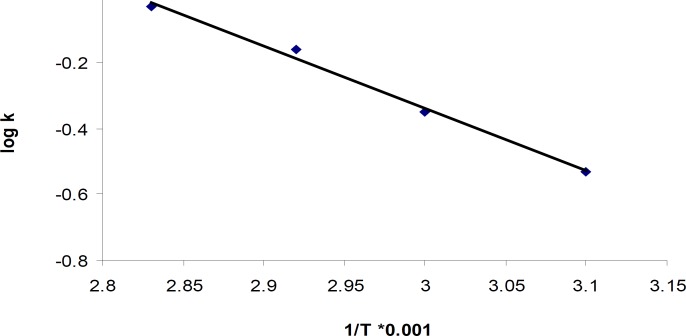
Arrhenius plot for degradation of cetirizine dihydrochloride in 0.5% H_2_O_2_

The results for degradation of cetirizine dihydrochloride in oxidative condition at four different temperatures were also shown in Figure 5. A linear relationship was observed between the log residual percent of cetirizine dihydrochloride versus time which indicates a pseudo-first-order kinetics. The values for the apparent first order rate constants and half-lives are presented in Table 5. In addition, by plotting the Arrhenius equation (Figure 6), the activation energy for the hydrogen peroxide mediated degradation was found to be 36.3 KJ/mole. 

**Table 5:** Degradation equation, apparent rate constant (k) and half-life (t_1/2_) for cetirizine dihydrochloride in 0.5% H_2_O_2_

**Temperature (**°**C)**	**Equation**	**r** ^2^ **-value**	**k (h** ^-1^ **)**	**t**_1/2_ ** (min)**
50	y = - 0.127x + 1.997	0.994	0.293	141.9
60	y = - 0.193x + 1.966	0.994	0.444	93.6
70	y = - 0.301x + 2.037	0.996	0.693	60.0
80	y = - 0.409x + 1.965	0.999	0.942	44.1
